# Unmanned aerial vehicle image detection of maize-YOLOv8n seedling leakage

**DOI:** 10.3389/fpls.2025.1569229

**Published:** 2025-05-23

**Authors:** Jiaxin Gao, Feng Tan, Jiapeng Cui, Zhaolong Hou

**Affiliations:** ^1^ College of Engineering, Heilongjiang Bayi Agricultural University, Daqing, China; ^2^ College of Information and Electrical Engineering, Heilongjiang Bayi Agricultural University, Daqing, China; ^3^ School of Mechanical Engineering, Chongqing Three Gorges University, Chongqing, China

**Keywords:** maize seedlings, unmanned aerial vehicle, natural scene, image processing, YOLOv8n

## Abstract

**Introduction:**

Missing seedlings is a common issue in field maize planting, arising from limitations in sowing machinery and seed germination rates. This phenomenon directly impacts maize yields owing to the poor effect of unmanned aerial vehicle (UAV) remote sensing images based on seedling leakage detection in fields. Therefore, this study proposed a method for detecting missing seedling in fields based on UAV remote sensing to quickly and accurately detect missing seedling and facilitate subsequent crop management decisions.

**Methods:**

The method calculates the rated inter-seedling distance in UAV-captured images of maize fields using a combination of image processing techniques, including background segmentation, stalk center region detection, linear fitting of plant rows, and average plant distance calculation. Based on these calculations, an improved Maize-YOLOv8n model was employed to detect actual seedling emergence.

**Results:**

The experimental results demonstrate that the new model achieved superior performance on a self-constructed dataset, with a mean average precision (mAP) of 97.4%, precision (P) of 94.3%, recall (R) of 93.1%, and an F1 score of 93.7%. The model was lightweight, comprising only 1.19 million parameters and requiring 20.2 floating-point operations per second (FLOPs). The inference time was 12.8 ms, satisfying real-time detection requirements. Performance evaluations across various conditions, including different leaf stages, light intensities, and weed interference levels, further indicated the robustness of the model. In addition, a linear regression equation was developed to predict the total number of missing seedlings, with model performance evaluated using the root mean squared error (RMSE) and mean absolute error (MAE) metrics.

**Discussion:**

The results confirm the ability of the model to accurately detect maize seedling gaps. This study can evaluate the quality of seeding operations and provide accurate information on the number of missing seedlings for timely replacement work in areas with high rates of missing seedlings. This study advances precision agriculture by enhancing the efficiency and accuracy of maize planting management.

## Highlights

- Achieved 97.4% mAP in maize seedling detection usingMaize-YOLOv8n.
- Reduced model parameters to 1.19M for real-time UAV maize detection.
- Improved maize seedling detection under varied light, weed, and growth conditions.
- Proposed a regression model to predict maize seedling gaps accurately.
- Enhanced precision agriculture by automating seedling replanting guidance.

## 1 Introduction

Maintaining uniformity and density in crop planting is essential for maximizing yield and quality in modern agricultural production. As a critical global food crop (Baier et al., 2023), maize is pivotal to ensuring food security and supporting the agricultural economy. However, challenges such as missing seeds during sowing or non-emergence after sowing can lead to reduced crop emergence and lower yields. Therefore, accurately assessing maize leakage is crucial for crop management decisions and subsequent timely replacement work in areas with high leakage rates. In existing agricultural practice, maize seedling deficiency is typically determined based on phased field inspection and experience judgment after planting, which are inefficient and subjective. As such, satisfying the requirements of precision agriculture using these methods is challenging. With advancements in digital image processing and deep learning (DL), image-based maize seedling detection has emerged as a more efficient and accurate solution. Crop counting techniques are broadly categorized into traditional digital image processing and DL-based methods.

Traditional digital image processing has long been integral to crop counting. These methods involve preprocessing images, segmenting crops from their backgrounds based on features such as color (Teixidó et al., 2012), texture, and shape, and applying techniques such as morphological operations, skeletonization, contour detection, and corner detection. For instance, in citrus counting, red–green–blue (RGB) images are converted into the hue–saturation–value (HSV) format, followed by threshold processing, orange detection, noise removal, watershed segmentation, and counting (Dorj et al., 2017). This algorithm shows promise for early yield prediction in individual citrus trees. Similarly, Wu et al. (2023) combined Deeplab V3+, a convolutional neural network (CNN) model, with classical image processing algorithms to segment banana bunches and calculate their number. They employed edge detection to extract banana finger centroids and clustering to determine the optimal bunch count, enabling intelligent decision-making for debudding timing. However, segmentation based on a single feature often has limitations. To address this issue, Sun et al. (2019) used a dual-threshold region growth algorithm that combines color and spatial features to segment cotton balls and developed three geometry-based algorithms for field yield estimation under natural light. In scenarios with uneven illumination and complex backgrounds, Malik et al. (2018) improved the HSV color space and watershed segmentation methods to detect ripe tomatoes with an accuracy of 81.6%. Liu et al. (2016) developed an automated method for counting wheat seedlings in the field using image processing, establishing a skeleton optimization method and achieving an average accuracy of 89.94% in overlapping areas. He et al. (2023) introduced an adaptive threshold method based on HSV color space to detect and locate Zanthoxylum fruit, outperforming fixed-threshold methods. However, traditional digital image processing methods are highly dependent on artificial design features such as color and texture and are not sufficiently robust in complex field scenes. The method based on color threshold segmentation is easily affected by illumination changes, resulting in over-segmentation or under-segmentation. Morphological manipulation is sensitive to the diversity of seedling shape and size, and adapting to maize seedlings of different leaf stages is challenging. In addition, the traditional method needs to manually adjust parameters for different scenarios, and the generalization ability is limited.

DL has significantly expanded the possibilities for crop detection and counting. DL object detection methods are typically employed for crop quantity estimation, and many researchers have refined existing models to enhance accuracy. Liu et al. (2020)integrated drone and smartphone imagery and employed models based on ResNet and Faster R-CNN (VGGNet). Their results demonstrated that using ResNet as the feature extraction network achieved a maximum accuracy of 95.95% in corn tassel detection, and after optimizing anchor sizes, the detection accuracy for high-resolution images increased to 89.96%. Alzadjali et al. (2021) developed two methods for detecting corn ears—TD-CNN and Faster R-CNN—based on drone images. Among these, Faster R-CNN achieved a superior F1 score of 97.9% compared to TD-CNN’s 95.9%, confirming the potential of deep learning to precisely capture corn ears and suggesting that further improvements in CNN architectures could enhance detection efficiency and adaptability. Xu et al. (2022) combined an improved Mask R-CNN with a YOLOv5x model to detect and count complex field corn leaves using drone imagery. The detection average precisions (AP) for fully unfolded and emerging leaves were 89.6% and 54.0%, respectively, validating the efficiency and potential of integrating drone imagery with deep learning for crop phenotyping. Niu et al. (2024) addressed the challenge of detecting drought stress in corn seedlings under climate change conditions by proposing a drone image analysis method based on an improved YOLOv8+ model. By compressing the model with a C2F-Conv module and incorporating CA attention and BiFPN architectures to enhance small object recognition, the method achieved an mAP@50 of 89.16% along with a real-time detection speed of 24.63 ms, thereby providing an effective solution for precise agricultural drought monitoring. Cui et al. (2023) introduced a real-time seedling counting method using improved YOLOv5s and ByteTrack algorithms, achieving 93.2% accuracy and a detection speed five times faster than manual methods. Jiang et al. (2024) proposed a cabbage detection algorithm based on YOLOv8-Cabbage, integrated with a positioning system using a Realsense depth camera, improving accuracy from 88.8% to 93.9%. Wang et al. (2024a) created a potato seedling dataset using unmanned aerial vehicle (UAV) imagery and proposed the VBGS-YOLOv8n model, which achieved an average accuracy of 98.4% compared with other state-of-the-art models. Wang et al. (2024b) proposed a method for detecting wheat seedlings using localized annotation, incorporating a space-to-depth conv module and a microscale detection layer to achieve a detection accuracy of 90.1%. Gao et al. (2024) developed YOLOv5-Tassel for detecting corn tassels in UAV imagery, achieving 98.70% accuracy through innovative processes such as attention mechanisms and multi-scale convolution. Zhu et al. (2024) introduced YOLOv5s-CEDB, a network for oil tea fruit detection, achieving an average mAP of 91.4% and an F1 score of 89.6% using deformable convolution and coordinated attention.

In recent years, crop detection technology based on UAV remote sensing and deep learning has rapidly advanced. Although these approaches have achieved promising results in crop detection and counting, studies directly targeting missing seedling detection remain scarce. Current mainstream object detection methods—such as YOLOv5, YOLOv8, and Faster R-CNN—still exhibit certain limitations. Specifically, detecting small objects in UAV images remains challenging, and the complex background information in field maize images—characterized by varying leaf stages, lighting conditions, and weed interference—can lead to both false positives and missed detections, ultimately compromising detection accuracy and the ability to count maize precisely. Furthermore, in order to satisfy mobile deployment requirements, the issue of model lightweighting must also be addressed. Based on these considerations, this study combines traditional image processing techniques with deep learning to propose a flexible and reliable algorithm for counting missing maize seedlings in field conditions.

In this study, a missing seedling detection method based on UAV remote sensing images, combined with an improved target detection algorithm and traditional digital image processing, was proposed. The method is robust and satisfies the real-time detection requirements for maize seedlings in different leaf stages, different light intensities, and degrees of weed interference in the field. SCConv modules were introduced into the backbone and neck of YOLOv8n to reduce the number of model parameters using a dual redundancy strategy while retaining the expression ability of key features. The BiFormer bidirectional sparse attention mechanism was embedded in the SPPF layer, and the global context information of maize seedlings was captured using a dynamic routing strategy, which significantly improved the detection robustness under weed interference scenarios. The multi-scale detection head was reconstructed, the large target detection branch was removed, and the small target detection scale was expanded, improving the detection accuracy of early three-leaf seedlings. Subsequently, the number of maize seedlings in the field can be accurately obtained through a series of traditional image processing methods such as background segmentation, stem center area search, and average plant distance calculation. Combined with the detection results of the detection algorithm, the information of missing seedlings can be effectively detected. Experimental verification revealed that this model can accurately detect corn seedlings under complex natural scenes. This study effectively detects the number of corn seedlings, provides a scientific basis and technical support for subsequent corn crop management decisions, and contributes to the technological advancement of agricultural production.

The main contributions of this study are as follows:

- Integrating traditional image processing methods with the Maize-YOLOv8n model for automated maize seedling gap detection.
- Employing advanced image processing techniques, including background segmentation, stalk center region identification, and average plant distance calculation, to determine maize seedling counts.
- Improving the mAP, recall (R), and F1 score of the Maize-YOLOv8n model while reducing model parameters, enabling efficient detection in complex field conditions.

The remainder of this paper is structured as follows: Section 2 details the structure and implementation of the algorithm, Section 3 presents experimental results and analysis, Section 4 discusses the findings, and Section 5 concludes the study.

## 2 Materials and methods

### 2.1 Workflow

**Figure 1:**
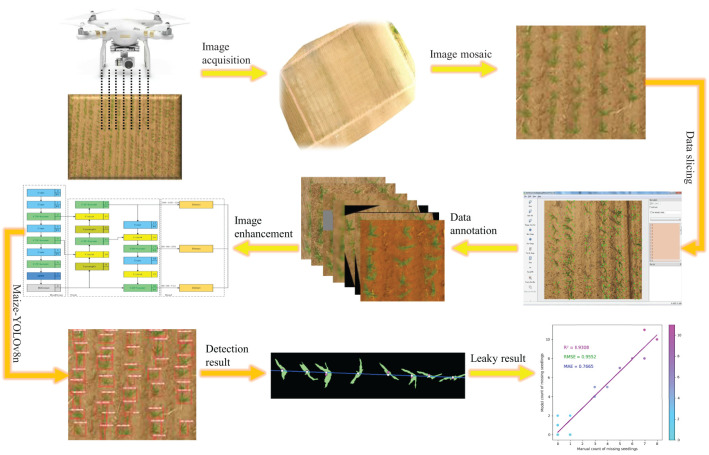
Missing seedling detection workflow.

The workflow of this study, as illustrated in **Figure 1**, comprises three primary components:

- Establishing an image dataset for maize leakage detection.
- Developing the Maize-YOLOv8n network structure to statistically analyze maize seedling counts.
- Creating a traditional image processing detection model for maize seedling count estimation.

### 2.2 Dataset creation

#### 2.2.1 Data collection

UAV image data were collected from June 12 to 20, 2022, between 10:00 and 13:00 daily at the Modern Agricultural Science and Technology Demonstration Park in Durbert Mongolian Autonomous County, Daqing City, Heilongjiang Province. The center of the park is located at 46°49’N, 124°26’E, with an elevation of approximately 147 m above sea level.

The maize varieties planted include Jinwanyu Z658, Tianyu 108, Meiya 81, Lihe 869, and Jindoctor 825, with a row spacing of 65 cm and a plant spacing of 25 cm. The study focused on maize grown in mechanically seeded plots. Data acquisition was conducted using a DJI Spirit 3P UAV equipped with a 1/2.3-inch complementary metal-oxide semiconductor image sensor. The sensor had an effective resolution of 12.4 Mp (total resolution: 12.76 Mp), and the camera was equipped with a lens offering a 94°field of view, a 20 mm focal length (35 mm equivalent), and an aperture of f/2.8.

**Figure 2:**
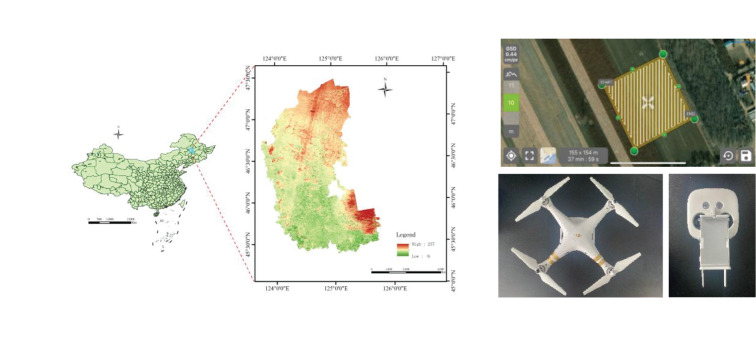
Test site and equipment design.

**Figure 3:**
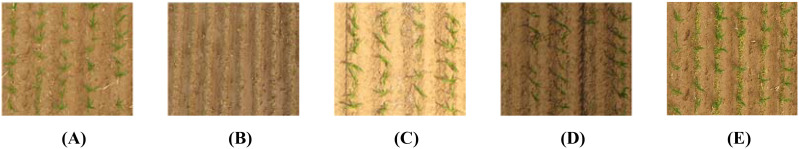
Data sample. **(A)** Maize seedling in three leaf stage. **(B)** Incipient maize seedlings with three leaves. **(C)** Maize seedlings under high light intensity. **(D)** Maize seedlings under low light intensity. **(E)** Maize seedlings accompanied by weeds.

The camera was positioned vertically downward during the operation. The flight height of the UAV should consider the image resolution and coverage area; a lower height provides a higher resolution but reduces the coverage area and increases the data acquisition workload. A higher height increases the coverage; however, the lower ground resolution captured by the camera will lead to blurred details and edges of corn seedlings in the image, reducing the detection accuracy of the model for corn seedlings. Considering the above factors, the UAV shooting height was set to 10 m, and the ground sampling distance was 0.44 pixels/cm. Images were acquired under stable solar radiation and clear, cloudless skies to minimize the loss of texture features owing to cloud cover. Route planning was performed using Pix4Dcapture, with the parallel and side overlap of the UAV set to 80% and 70%, respectively. Factors such as target coverage, field obstacles, and battery life were considered to optimize the flight path of the UAV. **Figure 2** illustrates the test site, route planning software, and acquisition equipment. Sample data collection is shown in **Figure 3**.

#### 2.2.2 Data annotation and partitioning

**Figure 4:**
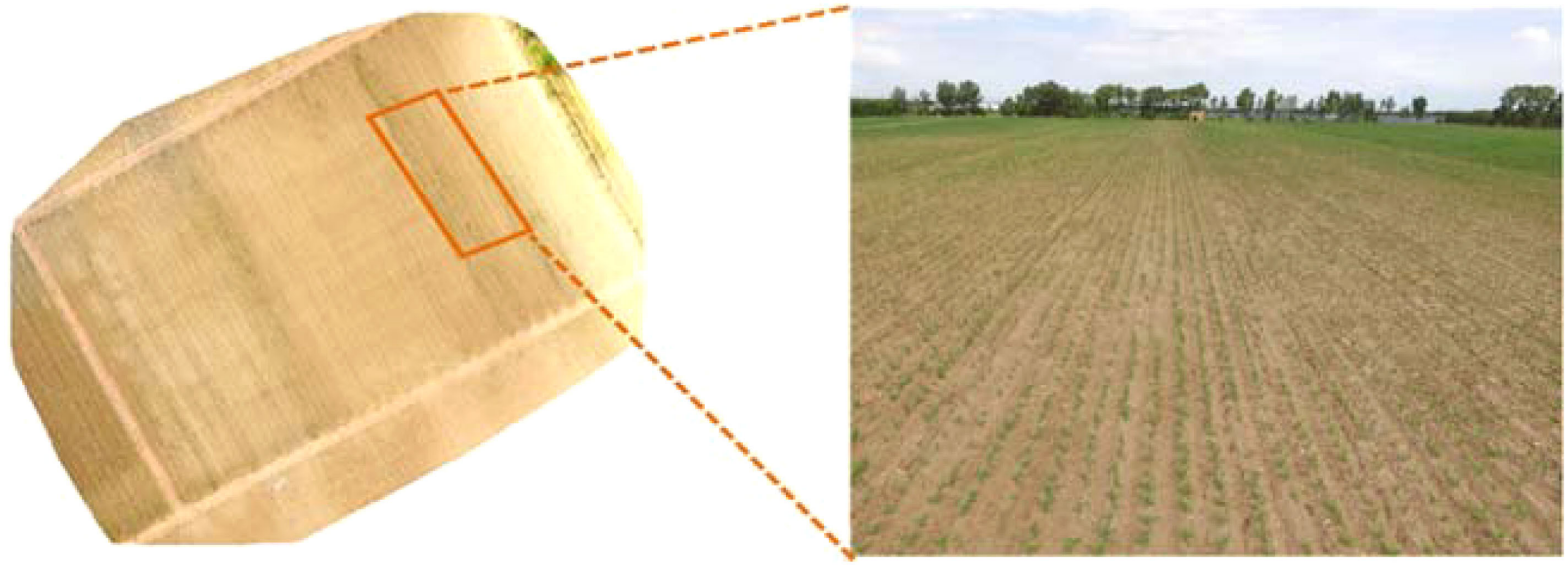
Image stitching example.

In total, 300 maize seedling images were collected during the experiment. These images were spliced into high-resolution maps using Pix4Dmapper (Song et al., 2016), as shown in **Figure 4**. Creating samples with sufficiently large areas without compromising the network detection speed was challenging owing to the large sizes of the spliced images and the dense packing of seedlings. However, this was addressed by slicing the images into smaller segments. The sliced images were screened to remove blurry or distorted samples, leaving 550 images for use in the dataset. Maize seedlings in the images were annotated using the LabelImg tool, which generated extensible markup language files containing ground-truth information. The dataset was then divided into training, validation, and test sets at a ratio of 7:1:2 (Zhao et al., 2024), with 385 images in the training set, 55 in the validation set, and 110 in the test set.

#### 2.2.3 Data enhancement

**Figure 5:**
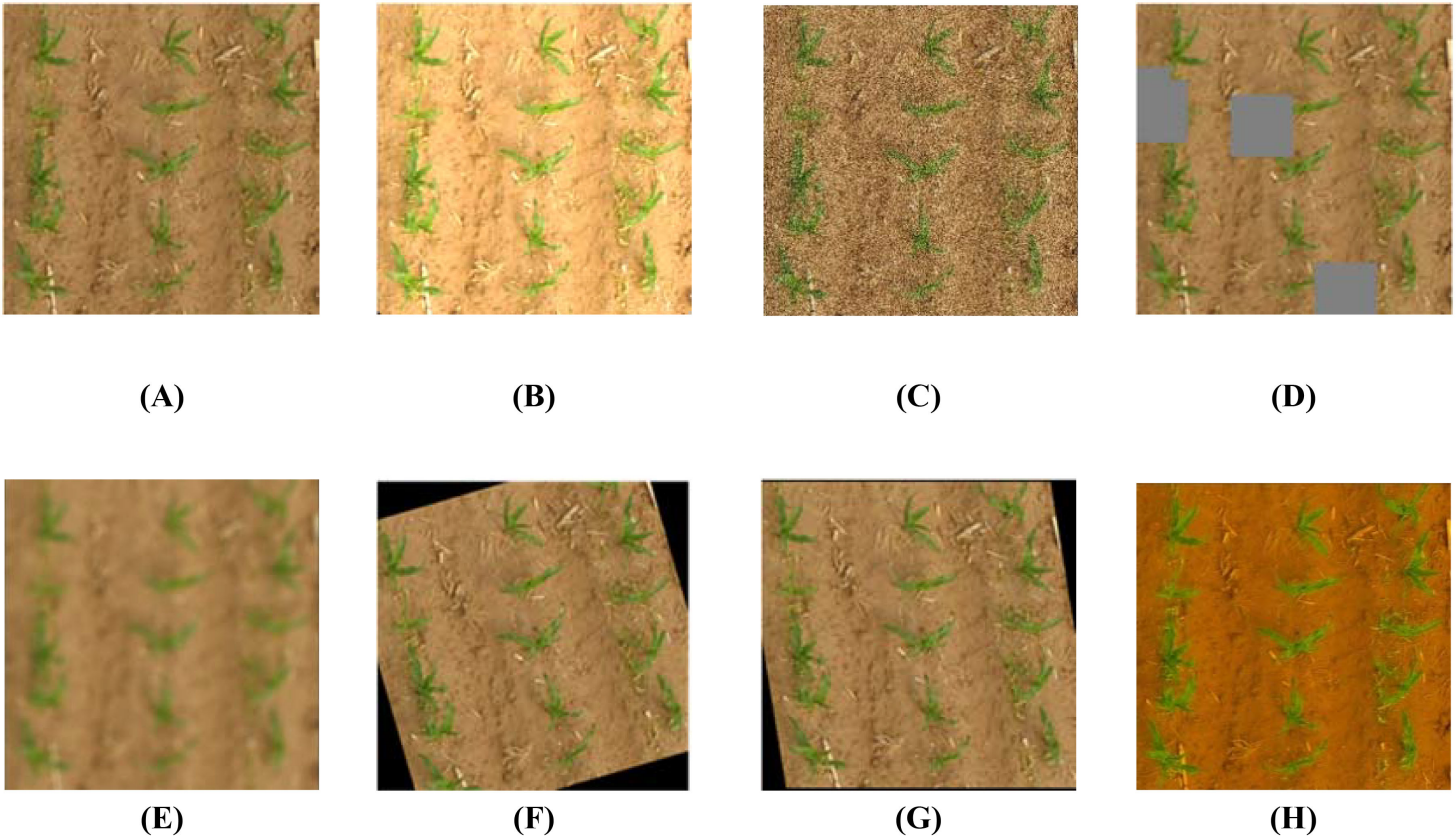
Data enhancement examples: **(A)** original image, **(B)** random brightness, **(C)** Gaussian noise, **(D)** random filling, **(E)** blur processing, **(F)** random rotation, **(G)** random tailoring, and **(H)** random saturation.

Data augmentation techniques were applied to the training set to improve the robustness and detection performance of the model (Rebuffi et al., 2021). Methods such as random filling, rotation, saturation adjustment, brightness, and cropping were used, along with the addition of Gaussian noise and blur processing (Shorten and Khoshgoftaar, 2019; Song et al., 2020). During augmentation, four enhancement methods were randomly selected for each image. After processing, 2,200 enhanced images were generated. The final training set included 1,540 images, with the test and validation sets containing 440 and 220 images, respectively. **Figure 5** illustrates the effects of the data augmentation process.

### 2.3 Maize-YOLOv8n seedling number detection model

This study developed a maize seedling counting model based on YOLOv8n, a cutting-edge and advanced version of the YOLO series, featuring enhanced performance and flexibility. YOLOv8 is well-suited for target detection tasks, as it was designed for speed, accuracy, and user-friendliness. However, despite its advancements, the detection accuracy of the model needs to be further improved, and its model parameters need to be reduced. In this study, the SCConv module was introduced into the C2f module of YOLOv8n, and the number of parameters of the model was reduced using the space-channel dual redundancy strategy, while the expression ability of key features was retained. The BiFormer module was added to the backbone network to focus the global context information of maize seedlings using a bidirectional sparse routing mechanism to significantly improve precision. The detection head size was reconstructed according to the small size of maize seedlings in the early stage, to improve the detection accuracy of three-leaf seedlings in the early stage.

#### 2.3.1 C2f-SCConv module

CNNs have achieved remarkable results in computer vision tasks; however, they often require substantial computational resources, partly owing to redundant feature extraction in convolutional layers. Strategies such as model pruning, distillation, and quantization, along with lightweight architectures like MobileNet (Howard et al., 2019), ShuffleNet (Zhang et al., 2018), and GhostNet (Han et al., 2020), aim to address this issue. This study builds upon these strategies by leveraging spatial and channel redundancies in CNN features through the SCConv module (Li et al., 2023).

SCConv is an advanced convolutional module that enhances feature learning by integrating spatial and channel dependencies. This integration allows the network to adapt its responses based on contextual information, enabling more refined feature extraction. SCConv comprises a spatial reconstruction unit (SRU) that reduces spatial redundancy by separating and reconstructing redundant features, followed by a channel reconstruction unit (CRU) that minimizes channel redundancy and reduces computational cost using split-transform and fusion strategies.

**Figure 6:**
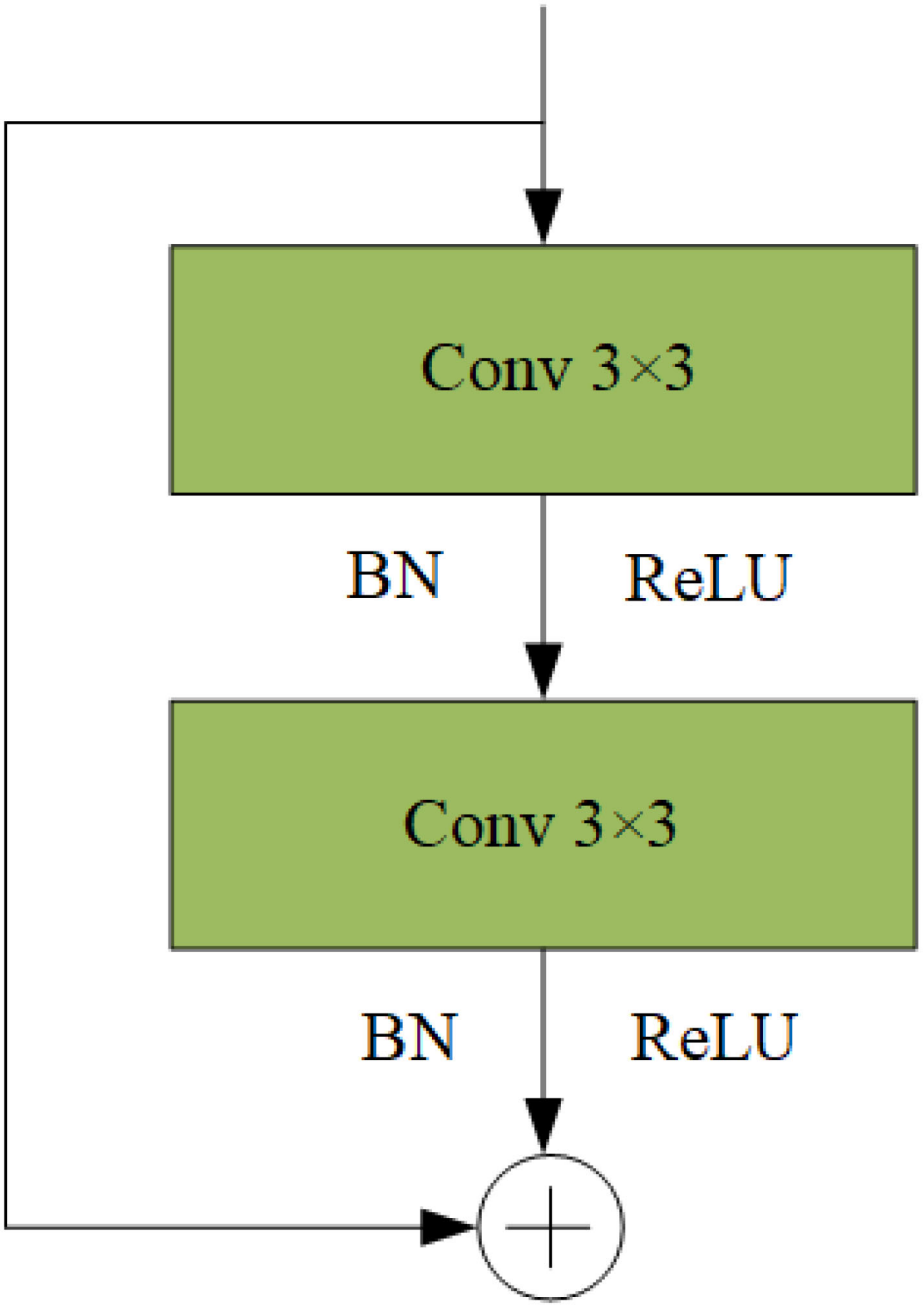
Bottleneck structure diagram.

**Figure 7:**
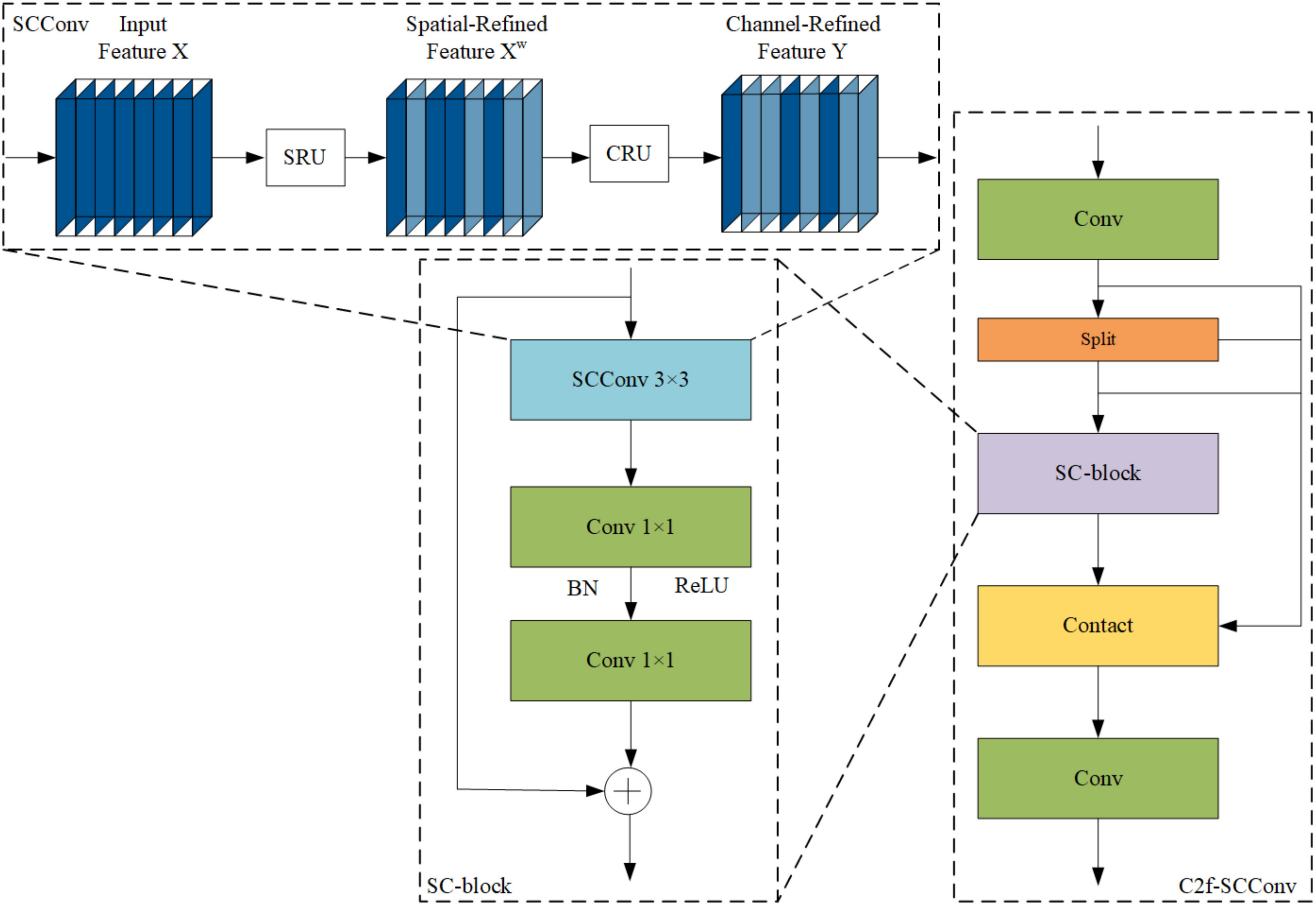
C2f-SCConv module structure diagram.

For an intermediate input feature X in the bottleneck residual block, the SRU generates a spatial refinement feature, XW, which the CRU then processes to yield the channel refinement feature, Y. By exploiting redundancies, SCConv reduces intermediate feature map redundancy while enhancing the feature representation of the CNN. The bottleneck module, used extensively with YOLOv8, facilitates the extraction and fusion of multi-dimensional features; however, it also contributes significantly to computational overhead. **Figure 6** illustrates the bottleneck structure. This was mitigated by designing the SCConv-based SC block to replace the bottleneck module in the C2f module, serving as a primary gradient flow branch. This substitution reduces the floating-point operations and computational load of the model. **Figure 7** shows the structure of the C2f-SCConv module, which integrates SCConv to streamline computation during the convolution process.

#### 2.3.2 BiFormer attention module

Traditional object detection methods often struggle to extract global context information, as they primarily focus on local features. This limitation hinders performance in complex scenes. Sparse attention mechanisms have been proposed to address this; however, they frequently face two challenges: fixed static modes that lack adaptability and shared sampling subsets of key-value pairs across all queries, which limits interference resolution. This study overcomes these challenges by utilizing BiFormer, a dynamic, query-aware sparse attention mechanism (Zhu et al., 2023). BiFormer filters irrelevant key-value pairs at a coarse level, retaining only relevant regions, and applies fine-grained token-to-token attention within these areas. This dual-level approach enhances the ability of the self-attention mechanism to capture global context while establishing effective correlations across image regions.

**Figure 8:**
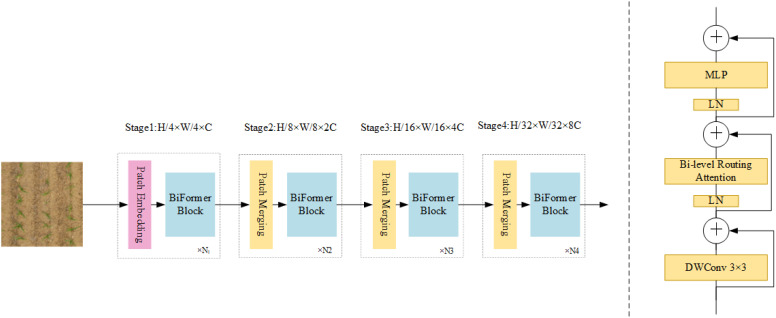
BiFormer attention module.

BiFormer enhances detection accuracy in the context of this study, where varying maize sizes, leaf stages, lighting conditions, and weed interference complicate seedling detection. By focusing adaptively on relevant areas, it effectively handles complex field environments. **Figure 8** shows the integration of the BiFormer module into the detection framework.

#### 2.3.3 Improved detection head

Detecting small targets, such as pre-trilobate stage maize seedlings, poses challenges for traditional YOLO networks, which tend to prioritize larger, more prominent targets on feature maps. We addressed this by providing an improved detection head (IDH), created by increasing the receptive field sizes from 80 × 80, 40 × 40, and 20 × 20 to 160 × 160, 80 × 80, and 40 × 40, respectively. This expansion improves the ability of the model to accurately detect small-sized seedlings.

**Figure 9:**
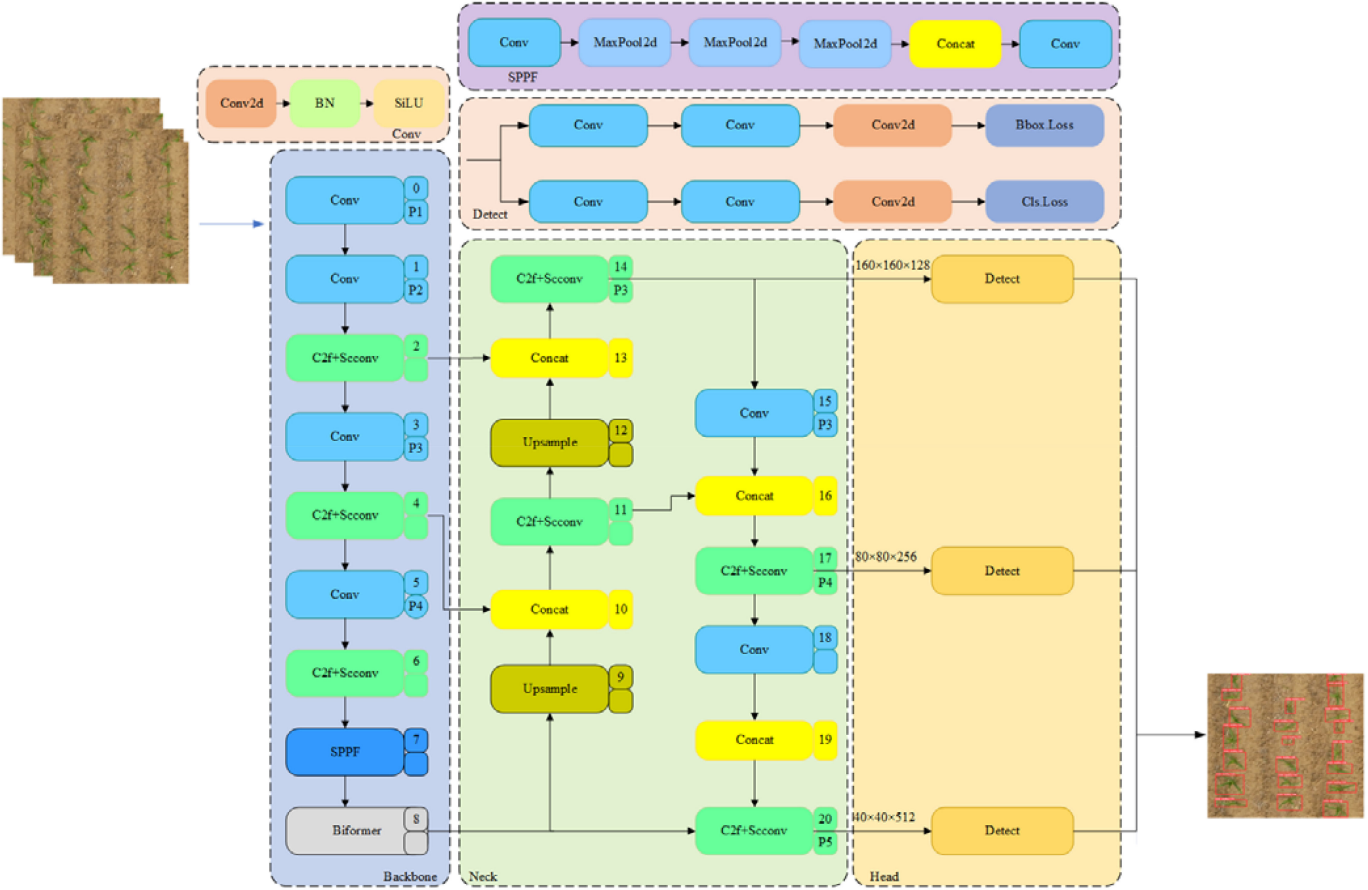
Structure of the Maize-YOLOv8n model.

The improved detection head also optimizes feature learning, enhancing sensitivity and recognition for small targets. Although the number of channels in the detection head was reduced, the remaining output channels were sufficient for detecting maize seedlings. This reduction in channels decreased computational cost and memory usage, accelerating both model training and real-time inference. By streamlining feature maps, the detection head achieves a balance between performance and efficiency, resulting in faster and more precise seedling detection in complex environments. **Figure 9** presents the network structure of the Maize-YOLOv8n model, showcasing the adjustments made to optimize its detection capability for small targets.

### 2.4 Counting seedlings

#### 2.4.1 Background segmentation

In field conditions, variations in lighting and interference from weeds can complicate maize seedling segmentation. Therefore, selecting an appropriate method for isolating green plants is crucial. This study employed the super green segmentation method to isolate maize seedlings from the background. First, the original RGB image was read, and the R, G, and B channels were separated. The green factor was then selected as the color feature to distinguish the green plants from the background. The green factor was calculated using Equation 1.


(1) ExG=2G−R−B.

**Figure 10:**
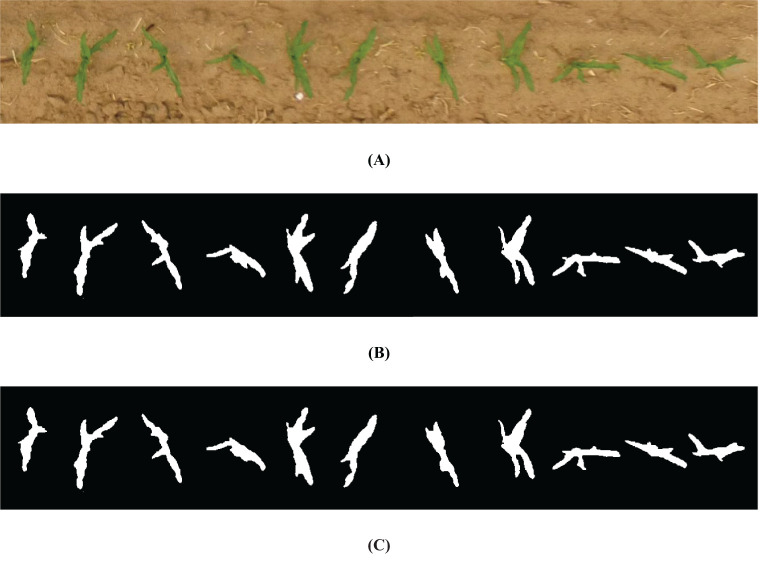
Background segmentation diagrams: **(A)** Original image, **(B)** binary image, and **(C)** images after morphological closure.

The green factor method is widely used in plant image recognition, as it suppresses shadows, dead grass, and soil, enhancing the prominence of green plant images for improved seedling recognition. After calculating the green factor, Otsu’s method was applied for image threshold segmentation. Otsu’s method automatically determines the optimal threshold values for green plant recognition, eliminating the need for manual parameter setting. This approach is convenient, stable, and effective, as demonstrated by the segmentation results shown in **Figure 10B**. The morphological closure method was applied to fill the black noise points within the white areas of maize seedling branches, addressing the small amounts of black noise in the branch junction areas of maize seedlings. The results are shown in **Figure 10C**.

#### 2.4.2 Stem center search

**Figure 11:**
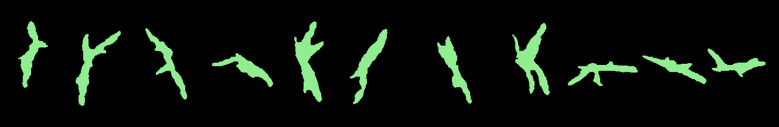
Outline of maize seedlings.

Accurately locating the centroid of each seedling is critical for determining the number of maize seedlings. First, a contour detection algorithm was used to identify the contours of each maize seedling in the image. The contours were further filtered to exclude smaller outlines, minimizing interference from weeds and non-maize seedlings. The remaining contours were considered to represent maize seedlings (**Figure 11**).

**Figure 12:**
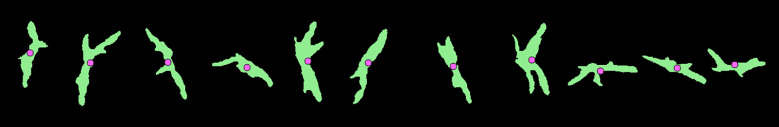
Centroid location map of maize seedlings.


The centroid coordinates of each maize seedling were calculated using the contour moments. This method ensures precise positioning, even in the presence of external noise. The centroid positioning results are shown in **Figure 12**. The centroid coordinates were determined using Equation 2.

(2) $$x_{0}=\frac{m_{10}}{m_{00}},y_{0}=\frac{m_{01}}{m_{00}}$$

where $m_{00}\;$ is the zero moment of the contour (area), $m_{10}$ is the first moment along the x-axis, and $m_{01}$ is the first moment along the y-axis. This calculation method is relatively insensitive to noise, ensuring accurate centroid determination under varying conditions.

#### 2.4.3 Plant alignment

Maize plants are typically arranged in distinct, straight rows, a feature that facilitates the determination of row directions based on the central positions of stalks. In this study, linear regression was employed to detect the row direction by minimizing a cost function. Given N data points $\left(x_{i},y_{i}\right)i=1,2,3\ldots N$ , representing the central positions of the stalks, the linear relationship can be expressed as Equation 3.


(3) $$y_{\left(x\right)}=y\left(x^{.},a_{1\ldots }a_{m}\right)$$

where $a_{1\ldots }a_{m}$ are the coefficients of the linear model. These coefficients were estimated using least squares regression, minimizing the value of Equation 4.


(4) $$\sum_{i=1}^{N}{\left[y_{i}-y\left(x^{.},a_{1\ldots }a_{m}\right)\right]}^{2}$$

**Figure 13:**
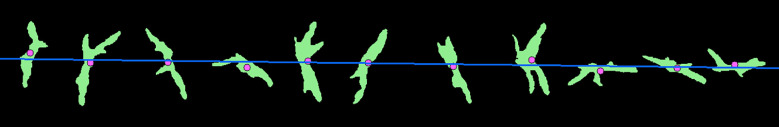
Maize seedling row straight line fitting results.

**Figure 14:**
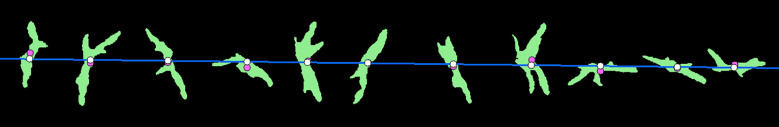
Plant center projection.

The distances between plants were calculated by projecting the plant centers onto the fitted line. The projection point was identified at the intersection of the vertical line from the plant centroid and the fitted line. **Figure 13** illustrates the line alignment fitting results, and **Figure 14** shows the projection points of plant centers.

#### 2.4.4 Average plant distance and seedling number

A set of plant distances, $D_{i}\left(i=1\ldots N\right)$ , was obtained to calculate the distances between consecutive projection points along the row direction. Minimum $\;D_{min}$ and maximum $D_{max}$ values in this set were determined, and a sequence $D_{j}\left(j=1\ldots M\right)$ was created by varying $D_{j}$ from $D_{min}$ to $D_{max}$ in steps of 10 pixels. For each $D_{j}$ , the absolute difference, $DT=ABS\left(D_{j}-D_{i}\right)$ , was calculated for every distance, $D_{i}$ , in the set. If DT was below a predefined threshold, the corresponding increment count, $CNT_{j}\left(j=1\ldots M\right)$ , was increased by one. After traversing all $D_{j}$ values, the value of $D_{t}$ in $D_{j}$ corresponding to the maximum $CNT_{j}$ was taken as the best estimate of the average plant spacing. The total number of seedlings was then calculated using Equation 5.


(5) $$Total=\sum_{k=1}^{n}round(\frac{D_{d}}{D_{t}})+1$$

where round() is the rounding function, $D_{d}$ is the spacing between the maximum and minimum vertical coordinates of each maize row, $D_{t}$ is the average plant spacing, and *k* is the number of rows. This approach provides a straightforward and accurate method for calculating the total number of maize seedlings per image.

## 3 Results and analysis

### 3.1 Experimental environment

The experiments were conducted using the PyTorch framework, and the details of the experimental environment are provided in **Table 1**. The input image size was set to 640 × 640 pixels. The model hyperparameters were configured as follows: the batch size was 16, the optimizer leveraged stochastic gradient descent with an initial learning rate of 0.01, the termination learning rate was 0.01, and the momentum parameter was set to 0.937. The learning rate was adjusted using the cosine annealing decay algorithm, with a decay coefficient of 0.0005.

**Table 1:** Test environment.

Configuration	Argument
CPU	Intel(R) Xeon(R) Platinum 8352V CPU @ 2.10 GHz
GPU	NVIDIA GeForce RTX4090 16G
RAM	120GB
Operating system	Ubuntu20.04
Accelerating environment	Cuda11.3 CUDNN 8.2.0
Development platform	PyCharm
Other	Numpy 1.17.0 Opencv 4.1.0

Training comprised 300 iterations, with weight files saved every 50 epochs. A log file was also generated to record the loss values for the training and validation sets. These hyperparameters were carefully selected to ensure faster convergence, minimize overfitting, and prevent the model from becoming stuck in local minima.

### 3.2 Model evaluation index

Several metrics were employed to objectively evaluate the performance of the model in detecting maize seedlings, including precision (P), recall (R), F1 score, average precision (AP), mean average precision (mAP), number of network parameters, floating-point operations per second (FLOPs) and inference time. For the experiments, the intersection over union threshold was set to 0.5. The formulas for calculating P, R, and F1 score are provided as follows Equations 6–8:


(6) $$Precision=\frac{TP}{TP+FP}$$

(7) $$Recall=\frac{TP}{TP+FN}$$

(8) $$F_{1}=\frac{2\cdot Precision\cdot Recall}{Precision+Recall}$$

where *TP* represents correctly detected (i.e., true positive) maize seedlings, *FP* denotes incorrectly classified (i.e., false positive) maize seedlings, and *FN* refers to missed detections (i.e., false negatives). The F1 score is the harmonic mean of precision and recall, with values closer to 1 indicating superior performance. AP is the area under the precision–recall (PR) curve, with higher values indicating better detection performance. Since this study focused on single-category detection (maize seedlings), mAP and AP are identical, as they both represent the area under the PR curve. mAP is calculated as Equation 9:


(9) $$mAP=\frac{1}{k}\sum_{i=1}^{k}AP_{i}$$

where *N* represents the number of categories. In addition, the number of network parameters, FLOPs and inference time were used to assess the complexity of the model.

### 3.3 Ablation test results

The ablation test results are presented in **Table 2**. The following key observations were made:

- Test 2: Incorporating the SCConv module into the C2f module reduced the computational complexity of the model. The number of model parameters decreased to 87.4% of the baseline network, FLOPs dropped to 7.2, and the inference time increased by 7.6 ms. This improvement also increased mAP by 1.9% and recall by 0.6%. This is because SCConv limits feature redundancy and enhances feature representation capability, improving the recall rate and average accuracy. However, although SCConv reduces the parameters and computation amount through spatial-channel feature reconstruction, the parallel efficiency decreases owing to the introduction of conditional branches and fine-grained operations, resulting in increased inference time.
- Test 3: The attentional mechanism module was added in experiment 3 based on the limitations in experiment 2, decreasing the precision of the model by 0.8%, improving the recall rate and mAP, and increasing the number of parameters, FLOPs, and inference time.
- Test 4: In experiment 4, after adding the BiFormer attention module on the basis of the baseline network, the accuracy rate, recall rate, and average accuracy of the model improved compared with the baseline network, and the precision rate reached the maximum of 95.2%, F1 also reached the maximum of 94.0%, and the calculation amount and complexity of the model increased slightly. The results showed that adding the BiFormer module improved the detection effect of maize seedlings, which might be because the BRA module was based on sparse sampling rather than undersampling and could retain fine-grained details.
- Test 5: In experiment 5, after improving the detection head, compared with the baseline network, the accuracy and F1 of the model decreased slightly, FLOPs increased slightly, all the evaluation indices of other models improved, and the number of model parameters decreased to 34.6% of the baseline network. This indicates that such improvements reduce the amount of computation required by the model.
- Test 6: Experiment 6 improved the detection head on the basis of experiment 4. The number of parameters decreased by 1.96M, the number of model parameters decreased to 43.5% of the baseline network, the precision rate, recall rate, and mAP all decreased, the model complexity increased, and the inference time improved.
- Test 7: In test 7, C2f-SCConv was replaced, and the detection head was improved. Compared with test 2, when only C2f-SCConv was replaced, the recall rate and mAP of the model improved slightly, and the precision rate and F1 decreased slightly. The number of model parameters reached the minimum of 0.92M, which was only 30.6% of the baseline model.

**Table 2:** Ablation test results.

Test	Baseline	C2f+SCConv	BiFormer	IDH	P (%)	R (%)	F_1_ score	mAP (%)	Parameters (M)	FLOPs (G)	Inference time(ms)
1	✓				94.6	91.0	92.8	94.2	3.01	8.1	5.4
2	✓	✓			94.3	91.6	92.9	96.1	2.63	7.2	13.0
3	✓	✓	✓		93.5	92.4	92.9	97.2	2.90	17.5	13.7
4	✓		✓		95.2	92.8	94.0	96.3	3.27	18.5	5.7
5	✓			✓	94.2	91.3	92.7	95.4	1.04	10.6	4.7
6	✓		✓	✓	94.5	91.9	93.2	95.5	1.31	21.0	5.2
7	✓	✓		✓	93.3	91.9	92.6	96.9	0.92	9.8	10.3
8	✓	✓	✓	✓	94.3	93.1	93.7	97.4	1.19	20.2	12.8

**Figure 15:**
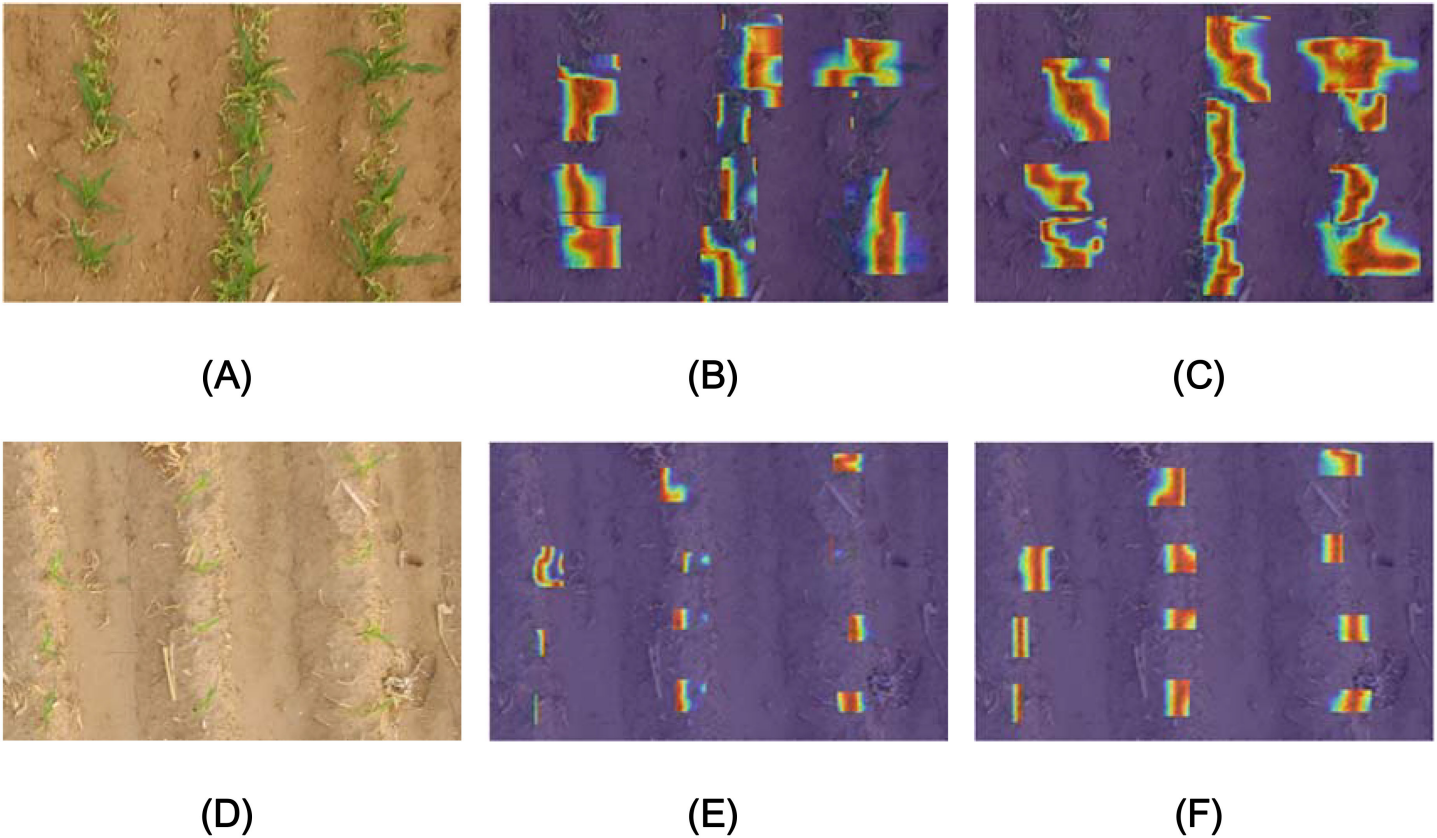
Heat map visualization of the model. **(A)** Original image, **(B)** YOLOv8n, **(C)** YOLOv8n+BiFormer, **(D)** Original image, **(E)** YOLOv8n, and **(F)** YOLOv8n+IDH.

Compared with the baseline network, the mAP of the improved Maize-YOLOv8n network increased by 3.2%, the recall rate increased to a maximum of 2.1%, the F1 score increased by 0.9%, the model precision 0.3%, and the number of model parameters shrunk to 39.5% of the baseline network. FLOPs were 12.1 higher, and the inference time was 7.4 ms slower. The above experiments demonstrate that Maize-YOLOv8n reduces the amount of calculation required by the model while P, R, F1 score, and mAP steadily increase. The results show that the model can ensure good detection and reduce the deployment cost. **Figure 15** shows that compared with the original model, the model with the BiFormer attention mechanism has a stronger feature extraction ability for maize seedlings in an environment with weeds. The original model had the issue of missed detection under the interference of weeds. The results show that the model can maintain high detection accuracy under weed interference. **Figure 15** shows that compared with the original model, the model with the small target detection head was more focused on detecting smaller seedlings. By contrast, the original model could not clearly detect light-colored seedlings. The model with the small target detection head demonstrated better attention in detecting corn seedlings, indicating that the small target detection head is crucial in small seedling detection in the pre-three-leaf stage. Consequently, the detection effect of the model on small targets was considered to have improved.

### 3.4 Visualization results of the receptive field heatmap

The receptive field (Ding et al., 2022) is a critical concept in CNNs, representing the region of an input image that a specific neuron can “perceive.” The size and characteristics of the receptive field significantly influence the performance, representation capabilities, and training efficiency of the model. Five well-trained models—YOLOv8n, YOLOv8n+C2f+SCConv, YOLOv8n+BiFormer, YOLOv8n+IDH, and Maize-YOLOv8n—were selected for comparison to support the analysis. A subset of 50 images from the validation set was resized to 1024 × 1024 pixels for testing. A 1024 × 1024 aggregated contribution score matrix was generated, in which each entry quantified the contribution of a pixel in the input image to the center point of the feature map produced by the final layer.

**Figure 16:**
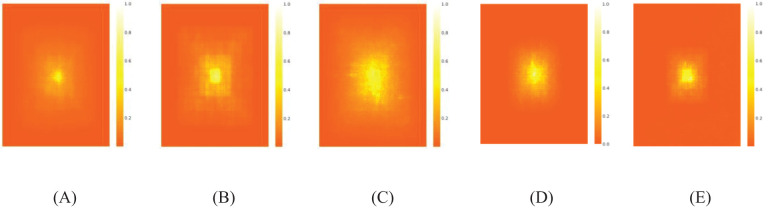
Visualization of the heat map of model receptive fields: **(A)** YOLOv8n, **(B)** YOLOv8n+C2f+SCConv, **(C)** YOLOv8n+BiFormer, **(D)** YOLOv8n+IDH, and **(E)** Maize-YOLOv8n.

As shown in **Figure 16**, the high-contribution pixels of YOLOv8n were concentrated around the central point, whereas the contribution of peripheral pixels was limited, indicating a constrained effective receptive field. YOLOv8n+C2f+SCConv exhibited higher contributions than Maize-YOLOv8n. The high-contribution pixels of YOLOv8n+BiFormer were more uniformly distributed, which explains its higher mAP. For YOLOv8n+IDH, the distribution of high-contribution pixels was highly concentrated, highlighting its improved focus on small target detection. Maize-YOLOv8n demonstrated higher peripheral contributions, indicating that it paid more attention to external elements.

Item *r* reflects the proportion of the smallest bounding rectangle that covers contributions above a given threshold, *t*. The high-contribution area ratios for YOLOv8n, YOLOv8n+C2f+SCConv, YOLOv8n+BiFormer, and YOLOv8n+IDH were 75.74%, 93.55%, 84.69%, and 28.72%, respectively. By comparison, Maize-YOLOv8n achieved a high-contribution area ratio of 97.21%, surpassing all other models. Therefore, most pixels contributed significantly to the final prediction.

### 3.5 Detection model comparison test

**Figure 17:**
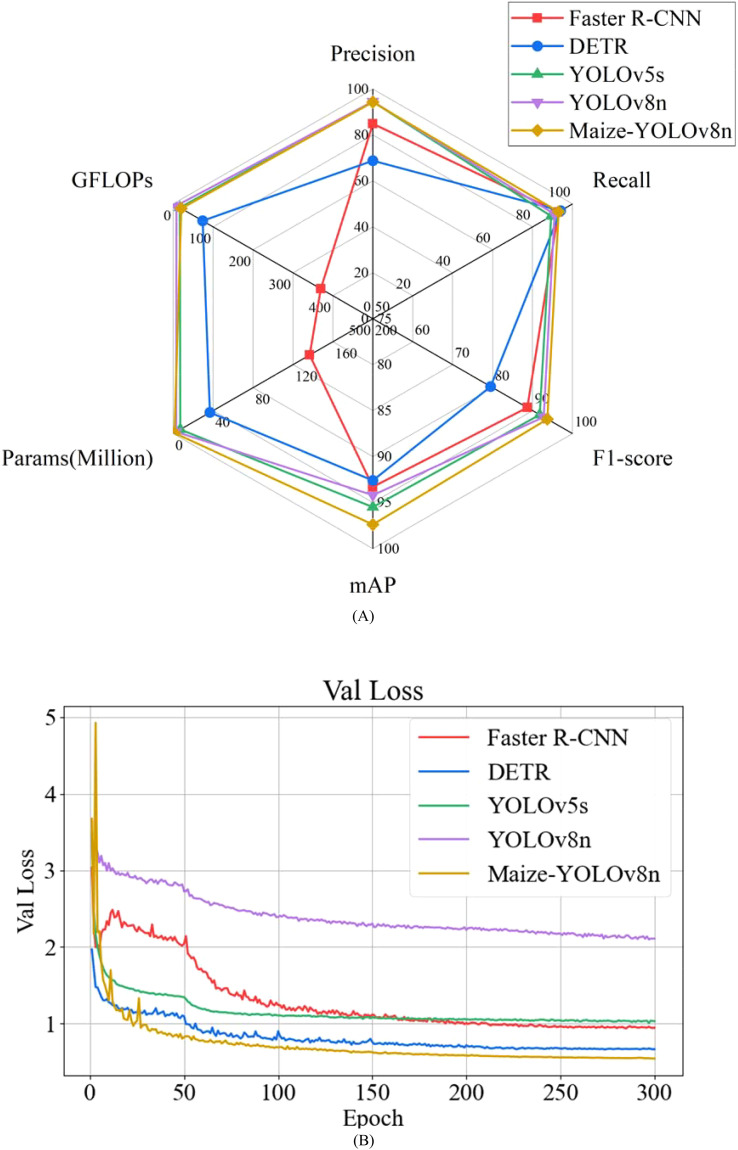
Performance comparison chart of different mainstream standard object detection models. **(A)** Comparison of precision, recall, F1 score, mAP, parameters, and GFLOPs. **(B)** Validation loss.

The Maize-YOLOv8n model, based on YOLOv8n, was compared with mainstream target detection network models, including Faster R-CNN, DETR, YOLOv5s, and YOLOv8n. The results are summarized in **Table 3**, with a radar map visualization shown in **Figure 17A** and the loss function curves of different detection models shown in **Figure 17B**.

**Table 3:** Comparison of the test results of maize seedlings with different models.

Model	P (%)	R (%)	F_1_ score (%)	mAP (%)	Parameters (M)	FLOPs (G)	Inference time (ms)
Faster R-CNN	84.9	92.9	88.7	93.3	136.68	369.7	9.8
DETR	68.8	94.0	79.5	92.6	36.74	73.6	25.8
YOLOv5s	94.5	89.3	91.8	95.5	7.06	16.5	9.4
YOLOv8n	94.6	91.0	92.8	94.2	3.01	8.1	5.4
Maize-YOLOv8n	94.3	93.1	93.7	97.4	1.19	20.2	12.8

**Table 3** reveals the following:

- Faster R-CNN: As shown in **Table 3**, this study first compares the two stages of target detection representing Faster R-CNN. Among the models compared, the Faster R-CNN model demonstrated the largest FLOPs of 369.7, which was 349.5G higher than the Maize-YOLOv8n model. This implies that the model requires a significant amount of computation. The P, R, F1 score, and mAP decreased by 9.4%, 0.2%, 5.0%, and 4.1%, respectively, compared with the Maize-YOLOv8n model. The Params were 136.68M, which was 135.49M higher than that of the Maize-YOLOv8n model. The inference time was faster than that of the Maize-YOLOv8n model.
- DETR: Second, the detection performance of Maize-YOLOv8n and the DETR model was compared. Compared with the Maize-YOLOv8n model, the P, F1 score, and mAP of the DETR model decreased by 25.5%, 14.2%, and 4.8%, respectively; the R increased slightly; and the Params, FLOPs, and inference time increased (36.74M, 73.6G, and 25.8 ms, respectively).
- YOLOv5s: Finally, the modeling effects of related models in the YOLO series were compared. Compared with the Maize-YOLOv8n model, the R, F1 score, and mAP of the previously proposed YOLOv5s model decreased by 3.8%, 1.9%, and 1.9%, respectively; P increased slightly; and FLOPs decreased by 3.7G. Although Params increased by 5.87M, the inference time was 3.4 ms faster.
- YOLOv8n: The R, F1, and mAP of the YOLOv8n model were 2.1%, 0.9%, and 3.2% lower than those of the Maize-YOLOv8n model, respectively; FLOPs decreased by 12.1G; the inference time was 7.4 ms faster; and P and Params increased by 0.3% and 1.82M, respectively.
- These results demonstrate that the Maize-YOLOv8n model outperforms similar detection models, achieving superior precision, recall, and mAP with fewer parameters (1.19M) and higher mAP (mAP of 97.4%). The model also offers faster detection speeds and lower computational complexity.

**Table 4:** Quantitative analysis of the high-contribution area ratio, r.

Test	Baseline	C2f+SCConv	BiFormer	IDH	t = 20%	t = 30%	t = 50%	t = 99%
1	✓				3.01%	5.28%	11.92%	75.74%
2	✓	✓			2.39%	4.32%	9.47%	93.55%
3	✓		✓		3.81%	6.33%	13.71%	84.69%
4	✓			✓	1.30%	2.11%	4.45%	28.72%
5	✓	✓	✓	✓	1.96%	2.84%	4.72%	97.21%

### 3.6 Detection performance in complex natural scenes

**Figure 18:**
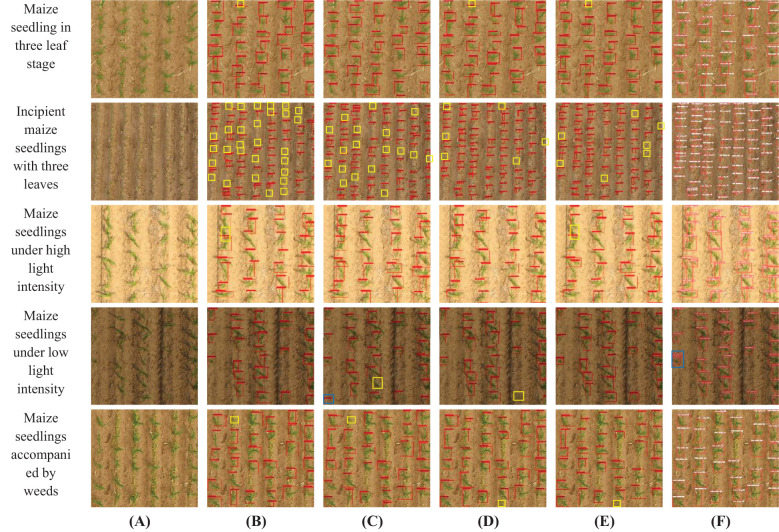
Examples of the maize seedling detection effects of five different models on different leaf stages, light intensity, and weeds. **(A)** Original image, **(B)** Faster R-CNN, **(C)** DETR, **(D)** YOLOv5s, **(E)** YOLOv8n, and **(F)** Maize-YOLOv8n.

This study evaluated the detection performance of the Maize-YOLOv8n model under various conditions, including different leaf stages, light intensities, and weed interference. The detection results were compared with the Faster R-CNN, DETR, YOLOv5s, and YOLOv8n models. In the test images shown in **Figure 18**, correct recognitions are shown in red boxes, unrecognized instances in yellow, and errors or repeated detections in blue.

**Figure 19:**
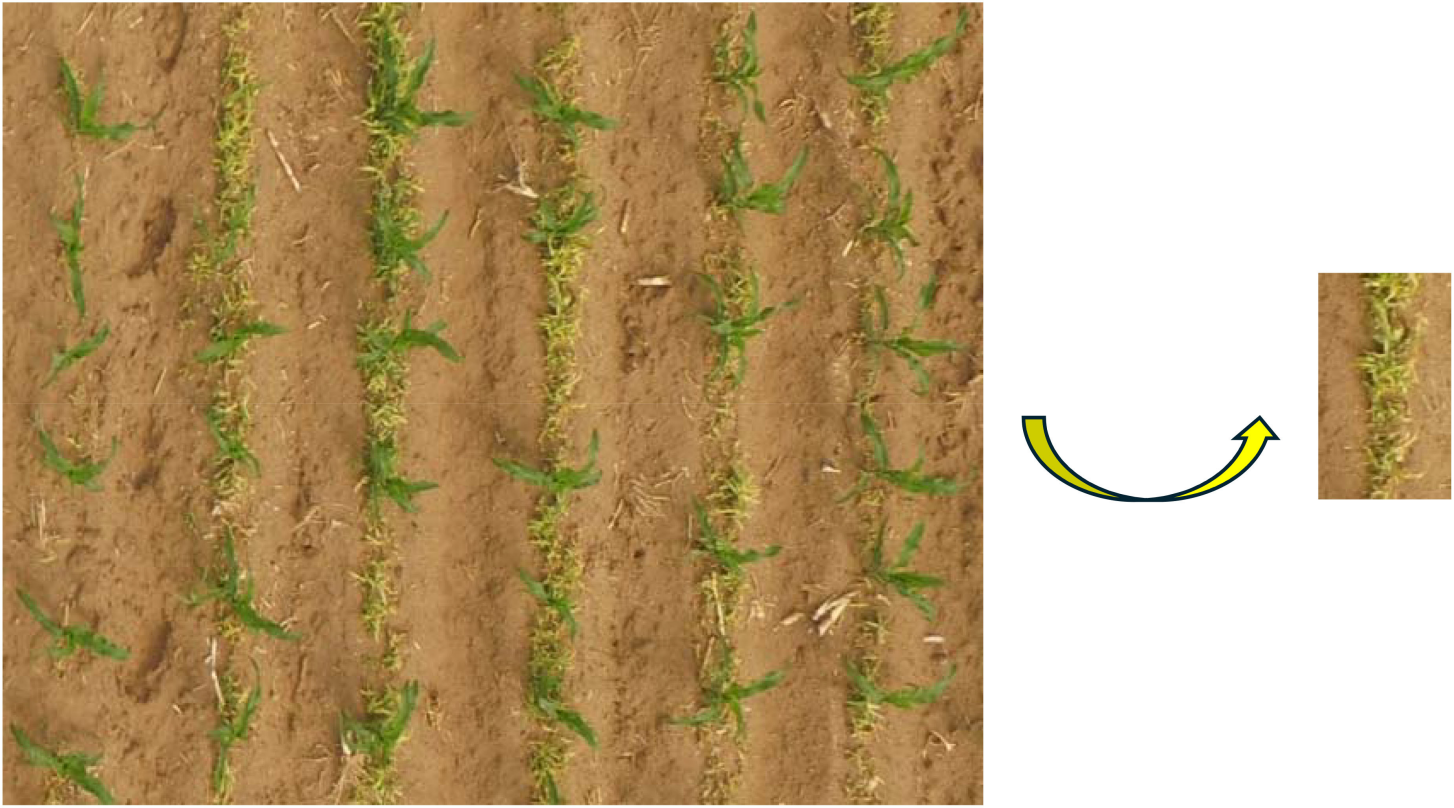
Barnyard grass.

**Figure 18** reveals the following:

- Leaf Stage: The images collected in this study included the early trilobate stage (approximately 10 cm tall) and late trilobate stage (approximately 15 cm tall) of maize seedlings. **Figure 18** demonstrates that Maize-YOLOv8n achieved higher detection rates for small seedlings in the early three-leaf stage, which are challenging to detect owing to their minimal size and low contrast with the ground. Although YOLOv5s and YOLOv8n performed better than Faster R-CNN and DETR, some detection errors persisted. The Maize-YOLOv8n model, with its enhanced detection head and BiFormer module, significantly reduced the number of undetected seedlings.
- Light Intensity: Faster R-CNN and YOLOv8n exhibited missed detections under high light conditions. Under low light, DETR and YOLOv5s faced similar issues, whereas Maize-YOLOv8n struggled with repeated detections at image edges owing to reduced contrast and blurred boundaries. Despite this, Maize-YOLOv8n demonstrated robust performance under medium and high light intensities.
- Weeds: Maize growth in the field environment is often accompanied by various weeds; the color and shape of weeds are similar to that of corn, which makes detection more challenging. The weed type observed in the collected images was barnyard grass, as shown in **Figure 19** below. Maize-YOLOv8n effectively detected all maize seedlings in the presence of weeds, including smaller seedlings at the edges, thus outperforming other models. Faster R-CNN and DETR failed to detect the seedlings among weeds, whereas YOLOv5s and YOLOv8n missed some smaller seedlings. Maize-YOLOv8n proved capable of detecting maize seedlings even amidst similar-colored weeds, ensuring stable detection.

In summary, Maize-YOLOv8n exhibited excellent performance across various leaf stages, light intensities, and weed conditions, making it well-suited to maize seedling detection and counting in complex field environments.

### 3.7 Complex natural environment experiment

**Figure 20:**
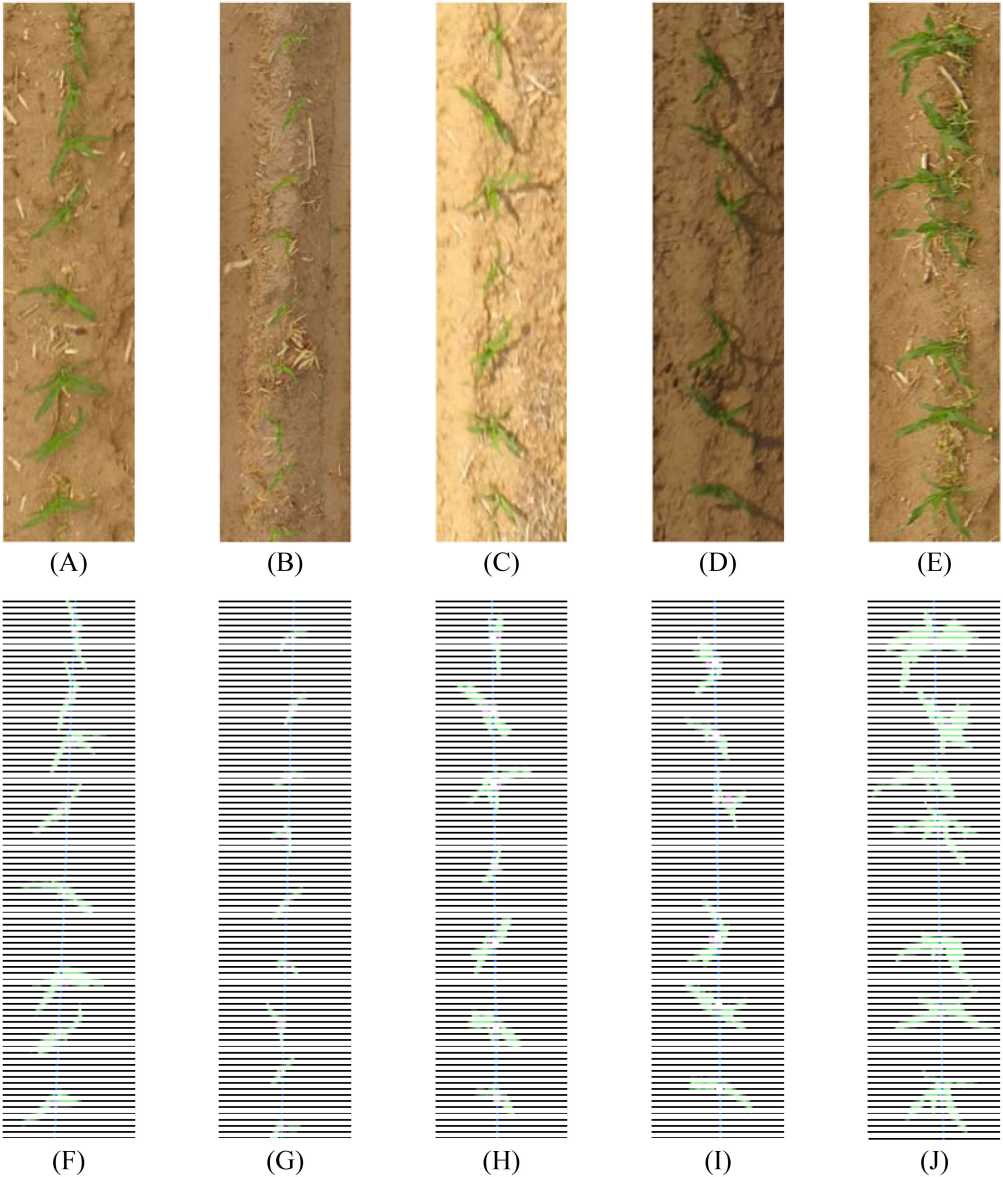
Effect of the seedling detection model on maize seedling detection at different leaf stages, light intensity, and weeds. **(A)** three-leaf stage, **(B)** early three-leaf stage, **(C)** high light intensity, **(D)** low light intensity, and **(E)** with weeds. Items **(F–J)** are effect diagrams representing items **(A, E)**, respectively.

In this study, traditional image processing algorithms were applied to count the number of maize seedlings in a field, with results shown in **Figure 20**. The algorithm effectively extracted maize seedlings under various conditions, including different leaf stages, light intensities, and weed interference. The test results for maize seedlings at the Three-leaf early seedlings, incipient seedlings with three leaves, seedlings under high light intensity, seedlings under low light intensity, and seedlings amidst weeds were 8, 9, 7, 6, and 7, respectively, matching the actual observed values.

### 3.8 Overall evaluation of the leaky seedling detection model

Nine plots were randomly selected from the experimental field to compare the detection performance of the proposed seedling leakage model with manual measurements. The differences between the manually detected and automated measurements were 19, 12, 15, 22, 14, 6, 11, 7, and 14 seedlings, respectively, as detailed in **Table 5**.

**Table 5:** Number of detected missing seedling in different plots.

Plots	Predicted leaked seedlings	Actual leaked seedlings	R^2^	RMSE	MAE
Plot 1	66	47	0.9308	0.9552	0.7665
Plot 2	81	69	0.9154	0.9800	0.7298
Plot 3	79	64	0.8000	1.5457	1.0924
Plot 4	86	64	0.7735	1.4523	1.1985
Plot 5	87	73	0.8509	0.9479	0.8203
Plot 6	69	63	0.8506	1.0114	0.8382
Plot 7	70	59	0.8655	1.0075	0.8523
Plot 8	83	76	0.9289	0.7413	0.6130
Plot 9	87	73	0.7650	1.4883	1.2705

**Figure 21:**
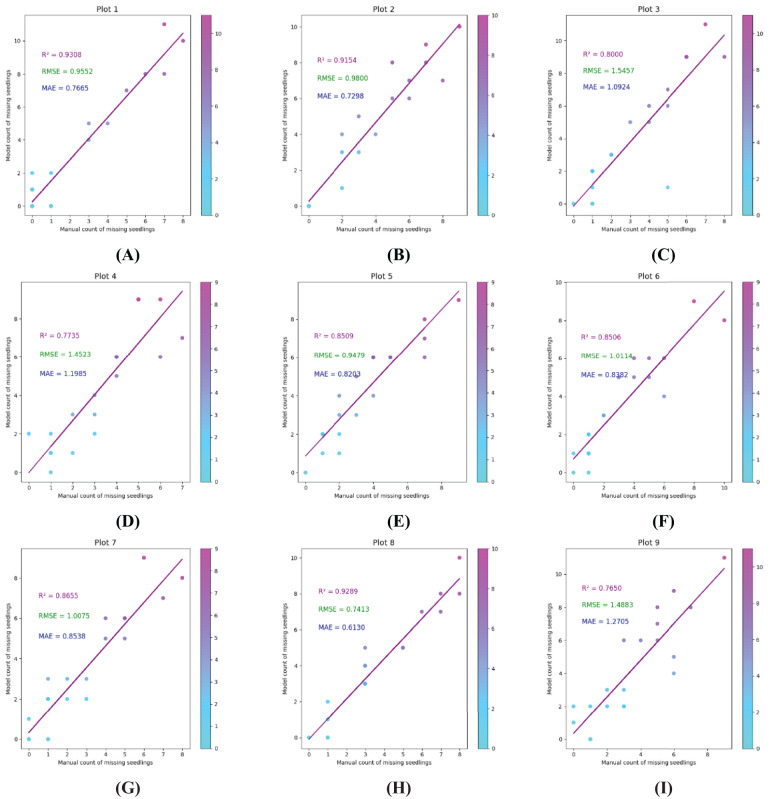
Accuracy verification of the algorithm on nine plots. Items **(A–I)** represent Plots 1–9, respectively.

The correlation between predicted and measured values was analyzed using RMSE and MAE metrics (**Figures 21A–I**).

The linear regression analysis of the predicted values yielded R^2^ coefficients of 0.9308, 0.9154, 0.8000, 0.7735, 0.8509, 0.8506, 0.8655, 0.9289, and 0.7650, respectively, for Plots 1–9. The RMSE values were 0.9552, 0.9800, 1.5457, 1.4523, 0.9479, 1.0114, 1.0075, 0.7413, and 1.4883, and the MAE values were 0.7665, 0.7298, 1.0924, 1.1985, 0.8203, 0.8382, 0.8523, 0.6130, and 1.2705. These results demonstrate that the proposed missing seedling detection model aligns closely with manual measurements, demonstrating its accuracy and efficiency in identifying seedling leakage.

## 4 Discussion

### 4.1 Resource identification initiative

The performance of the proposed maize seedling detection model was compared with those of similar studies. For example, Zhao et al. (2023) proposed LW-YOLOv7, a lightweight model achieving a mAP of 93.2%, precision of 89.3%, recall of 85.5%, and a parameter count of 59.4M. By contrast, the proposed Maize-YOLOv8n model achieved an mAP of 97.4%, precision of 94.3%, and recall of 93.1%, which is 4.2%, 5% and 7.6% higher than that of LW-YOLOv7, respectively, and the number of parameters is reduced by 58.21M to 1.19M. This detection accuracy improvement, coupled with a reduced computational load, satisfies real-time application requirements and facilitates deployment on portable and embedded devices in agricultural settings.

Mota-Delfin et al. (2022) evaluated YOLOv4, YOLOv4-TINY, and YOLOv5 structures for maize plant detection, with YOLOv5s achieving the best performance at 73.1% mAP. By comparison, the Maize-YOLOv8n model in this study significantly outperformed YOLOv5s, achieving 24.3% higher mAP. In addition, traditional image processing techniques were used in conjunction with Maize-YOLOv8n to compute maize emergence and leakage rates, providing a more comprehensive and precise assessment of maize seedling populations. The R² values for the nine test plots ranged from 0.7650 to 0.9308, indicating a strong correlation between the predicted number of missing seedlings and the actual number. These values indicate that the model is very accurate in predicting missing seedlings, especially in plots with more uniform planting patterns. However, we also acknowledge that the R² values of some plots can be improved further, especially in more complex or vegetated areas. The RMSE and MAE values range from 0.7413 to 1.5457 and from 0.6130 to 1.2705, respectively. These errors are small relative to the total number of seedlings per plot, indicating that the prediction of the model is acceptable in practical applications.

### 4.2 Environmental analysis

This study also analyzed the performance of the proposed model under complex natural conditions, comparing it with those of similar studies. Quan et al. (2019) employed a Faster R-CNN model with VGG19 to distinguish maize seedlings from weeds under varying weather and lighting conditions, achieving 97.71% precision. However, the weed detection performance was not evaluated. Yang et al. (2024) enhanced weed detection precision but did not test the model under varying light conditions or leaf stages. Lu et al. (2023) validated the performance of YOLOv5 under weedy environments, occlusion, and various growth stages but did not discuss the impact of different lighting conditions. In addition, for the irregular plots, we divided the irregular plots into rectangular sub-regions and applied the line detection algorithm one by one. In this way, our detection method can be applied to different shapes of farmland.

By contrast, this study evaluated the performance of the Maize-YOLOv8n model under diverse conditions, including different leaf stages, light intensities, and weed interference. The results demonstrate that the Maize-YOLOv8n model effectively adapts to complex natural scenes, highlighting its robustness and suitability for field applications.

### 4.3 Limitations and solutions

Two primary limitations were faced in this study. First, the mAP of the Maize-YOLOv8n model was 97.4%, indicating some detection errors. Field environments introduce challenges such as low image resolution, light condition variations (e.g., shadows, overexposure), and noise interference, all of which reduce feature extraction robustness. In addition, detection performance depends heavily on high-quality labeled datasets. Inaccurate annotations can lead to suboptimal feature learning, impacting detection accuracy. Finally, although the proposed model performs well on the test set, its performance may be limited by the singleness of the dataset because the dataset in this study was collected at the same place and under similar environmental conditions. The above problems can be addressed by optimizing the dataset by increasing diversified data sampling, improving the data annotation quality, and further collecting data under different regional and environmental conditions to improve the robustness and generalization ability of the model to complex field environments, enabling better adaptation to practical application scenarios.

Second, the regression analysis revealed suboptimal R^2^ coefficients and RMSE values for some plots, indicating seedling count inaccuracies. These errors primarily stem from challenges in distinguishing maize seedlings from weeds in densely vegetated areas, leading to plant distance miscalculations and seedling count overestimations. Future work will resolve these issues by incorporating additional morphological parameters, such as maize seedling skeleton pixels (Liu et al., 2022), to improve seedling count accuracy. Further refinements to the missing seedling detection algorithm will also be pursued to enhance performance in complex field environments.

## 5 Conclusion

In this study, the images of the maize seedling stage in the field environment were collected using the UAV remote sensing method. Subsequently, the maize seedling deficiency detection dataset was developed. The target detection method combined with traditional image processing was proposed to detect the missing seedling information accurately.

(1) The lightweight attention network (Maize-YOLOv8n) was designed. SCConv and BiFormer modules ensured model detection precision, significantly reduced the number of model parameters, and improved the precision of model detection in complex environments; the multi-scale detection head optimization strategy was proposed to solve the problem of missed detection of small targets, and the detection effect of early three-leaf seedlings was significantly improved. The fusion framework of DL and traditional image processing was developed, and the number of missing seedlings was accurately detected by dynamically calculating the plant distance and line direction fitting, which provides a new technical path for precision agriculture.

(2) The experimental results revealed that the mAP, R, and F1 score of our proposed Maize-YOLOv8n method on the test set were 97.4%, 93.1%, and 93.7%, respectively, and the P was 94.3%. In terms of the model parameters and FLOPs, the Maize-YOLOv8n also performed well, and the number of model parameters was only 1.19M, the FLOPs was 20.2, and the inference time was 12.8ms, which can satisfy the requirements of real-time model detection. Compared with the baseline network model, the mAP of Maize-YOLOv8n increased by 3.2%, the recall rate reached the maximum increase of 2.1%, the number of model parameters decreased to 39.5% of the baseline model, and the FLOPs increased by 12.1. The R^2^ coefficient of the determining coefficient between the number of missed seedlings predicted by the model and the actual number of missed seedlings was between 0.9308 and 0.7650. The RMSE was between 1.5457 and 0.7413. The MAE ranged from 1.2705 to 0.6130. Through experimental analysis, the model can be applied to maize leakage detection.

The proposed method can effectively reduce the manual labor intensity of maize leakage detection and provide guidance for the timely replacement of seedlings in areas with a high leakage rate in the later period, contributing to the technological advancement of the maize planting industry.

## Data Availability

The datasets presented in this article are not readily available because It can be made public in appropriate circumstances. Requests to access the datasets should be directed to Jiaxin Gao,a441380540@163.com.
